# ADP Regulates SNF1, the *Saccharomyces cerevisiae* Homolog of AMP-Activated Protein Kinase

**DOI:** 10.1016/j.cmet.2011.09.009

**Published:** 2011-11-02

**Authors:** Faith V. Mayer, Richard Heath, Elizabeth Underwood, Matthew J. Sanders, David Carmena, Rhonda R. McCartney, Fiona C. Leiper, Bing Xiao, Chun Jing, Philip A. Walker, Lesley F. Haire, Roksana Ogrodowicz, Stephen R. Martin, Martin C. Schmidt, Steven J. Gamblin, David Carling

**Affiliations:** 1 MRC Clinical Sciences Centre, Cellular Stress Group, Hammersmith Hospital Campus, Imperial College, DuCane Road, London W12 0NN, UK; 2 MRC National Institute for Medical Research, The Ridgeway, Mill Hill, London NW7 1AA, UK; 3 Department of Microbiology and Molecular Genetics, University of Pittsburgh School of Medicine, Pittsburgh, PA 15261, USA

## Abstract

The SNF1 protein kinase complex plays an essential role in regulating gene expression in response to the level of extracellular glucose in budding yeast. SNF1 shares structural and functional similarities with mammalian AMP-activated protein kinase. Both kinases are activated by phosphorylation on a threonine residue within the activation loop segment of the catalytic subunit. Here we show that ADP is the long-sought metabolite that activates SNF1 in response to glucose limitation by protecting the enzyme against dephosphorylation by Glc7, its physiologically relevant protein phosphatase. We also show that the regulatory subunit of SNF1 has two ADP binding sites. The tighter site binds AMP, ADP, and ATP competitively with NADH, whereas the weaker site does not bind NADH, but is responsible for mediating the protective effect of ADP on dephosphorylation. Mutagenesis experiments suggest that the general mechanism by which ADP protects against dephosphorylation is strongly conserved between SNF1 and AMPK.

## Graphical abstract

**Graphical Abstract:**

**Figure:**
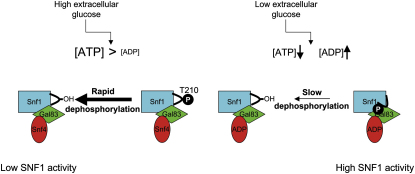

**Highlights:**

► ADP regulates SNF1 by protecting against dephosphorylation of Thr210 ► Snf4 binds two molecules of adenine nucleotide ► NADH binds to site 4 within Snf4, but does not compete with ADP protection ► Mechanism for ADP protection is conserved between SNF1 and AMPK

## Introduction

Glucose repression is the process whereby high levels of this sugar repress the expression of specific genes (Gancedo, 1992). In the budding yeast *Saccharomyces cerevisiae*, glucose depletion leads to activation of the SNF1 complex, which plays an essential role in derepression of glucose-repressed genes (Celenza and Carlson, 1986). SNF1 is the yeast homolog of mammalian AMP-activated protein kinase (AMPK). In mammals, activation of AMPK during conditions of energy stress, when intracellular ATP levels fall, leads to increased ATP generation and decreased ATP consumption (Carling, 2004; Kahn et al., 2005), and recent work has implicated it as a potential target for therapeutic intervention in metabolic diseases (Lage et al., 2008; Zhang et al., 2009). In budding yeast, SNF1 is essential for the adaptation of yeast for growth on low-glucose or on nonglucose carbon sources, such as sucrose (Celenza and Carlson, 1986). SNF1 is required for increased transcription of genes involved in metabolism of nonglucose carbon sources (Celenza and Carlson, 1986) and for a number of other processes, including sporulation, glycogen storage, and peroxisome biogenesis (Hardie et al., 1998). SNF1, like AMPK, is a heterotrimeric complex, consisting of a catalytic subunit (Snf1 in yeast; α in mammals) and two regulatory subunits (Sip1, Sip2, or Gal83 in yeast; β in mammals and Snf4 in yeast; γ in mammals) (Hardie, 2007; Hardie et al., 1998). SNF1 activity requires phosphorylation of a threonine residue in the activation loop (T-loop) of the kinase subunit (Thr210) (McCartney and Schmidt, 2001), and three protein kinases have been identified that phosphorylate and activate SNF1: Sak1, Elm1, and Tos3 (Hong et al., 2003; Sutherland et al., 2003). Lowering the concentration of extracellular glucose leads to the rapid activation of SNF1 (Wilson et al., 1996; Woods et al., 1994), correlating with increased phosphorylation levels of Thr210 resulting from a greatly reduced rate of Thr210 dephosphorylation (Rubenstein et al., 2008). In contrast, the activities of the upstream kinases in the cascade remain unaffected by a fall in glucose levels (Rubenstein et al., 2008). These findings suggest that activation of SNF1 is controlled at the level of the dephosphorylation step. This is similar to a mechanism previously described for the regulation of mammalian AMPK, where AMP protects against dephosphorylation of Thr172 by protein phosphatases (Davies et al., 1995; Sanders et al., 2007; Suter et al., 2006). Despite numerous studies, the identity of the metabolic signal that regulates SNF1 activity in response to glucose availability has remained elusive. Previous studies have shown that SNF1 is not regulated by AMP, either via an allosteric effect (Mitchelhill et al., 1994; Wilson et al., 1996; Woods et al., 1994) or by protecting it from dephosphorylation (Sanders et al., 2007).

## Results

### ADP Protects against Dephosphorylation of Thr210 in SNF1

**Figure 1:**
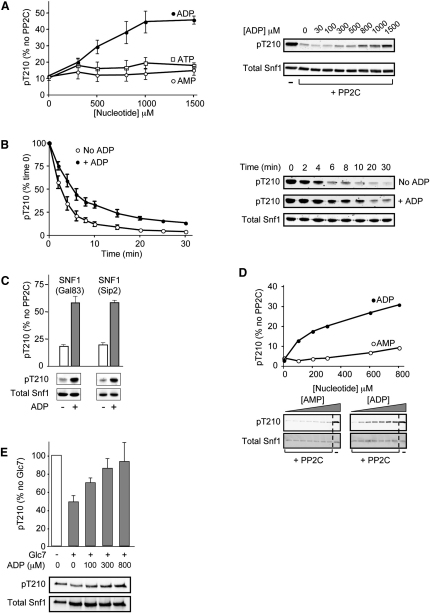
ADP Protects against Dephosphorylation of Thr210 in SNF1 (A) Recombinant SNF1 (Snf1_(1-633)_Gal83_(1-417)_Snf4_(1-322)_) was phosphorylated with CaMKKβ and incubated in the presence or absence of recombinant PP2Cα (26 ng) with increasing concentrations (0–1500 μM) of either AMP, ADP, or ATP. After incubation for 10 min at 37°C, the reactions were stopped by the addition of SDS sample buffer. Thr210 phosphorylation and total Snf1 levels were determined by western blot analysis and quantified using the LI-COR Odyssey infrared imaging system. Results shown are the mean ± SEM (n = 4 independent experiments) and are plotted as the ratio of P-Thr210:total Snf1 as a percentage of the control (in the absence of PP2C). Representative blots from an experiment using ADP are shown. (B) Active SNF1 was incubated at 37°C with PP2C in the presence or absence of ADP (800 μM). Aliquots were removed at the indicated times, and the reaction was stopped by addition of SDS sample buffer. Thr210 phosphorylation was determined as above, and the results shown are the mean ± SEM (n = 5 independent experiments). Representative blots from a single experiment are shown. (C) Recombinant SNF1 complexes comprised of either Snf1Gal83Snf4 or Snf1Sip2_(154-415)_Snf4 were phosphorylated with CaMKKβ and incubated with PP2C in the presence or absence of ADP (800 μM) for 10 min at 37°C. Thr210 phosphorylation was determined for each complex and plotted as the ratio of P-Thr210:total Snf1 as a percentage of the control value (measured in the absence of PP2C). Results shown are the mean ± SEM (n = 4 independent experiments), and a representative blot is shown for each complex. (D) SNF1 complexes purified from yeast grown in the presence of sucrose as the carbon source were incubated with PP2C in the presence of increasing concentrations (0–800 μM) of either AMP or ADP. After incubation for 10 min at 37°C, the reactions were stopped by the addition of SDS sample buffer, and the level of Thr210 phosphorylation and total Snf1 were determined by western blotting. Data from a single experiment together with the corresponding blots are shown. The level of Thr210 phosphorylation in a control incubation lacking PP2C is shown in each case, and the dashed line indicates the removal of intervening lanes from the samples run on the same gel. (E) TAP-tagged Glc7 was purified from yeast cells and used to dephosphorylate native SNF1 complexes in the presence or absence of varying concentrations of ADP for 10 min at 37°C. Thr210 phosphorylation was determined and plotted as the ratio of P-Thr210:total Snf1 as a percentage of the control value (measured in the absence of Glc7). Representative blots showing Thr210 phosphorylation and total Snf1 are shown below the graph.

We reported recently that ADP protects AMPK from dephosphorylation (Xiao et al., 2011). In order to investigate the effect of ADP on SNF1, we purified the heterotrimeric SNF1 complex (Snf1, Gal83, Snf4, which represents the predominant form of SNF1 in vivo) following expression in *E. coli* and activated it in vitro by phosphorylation with calcium/calmodulin-dependent protein kinase kinase β (CaMKKβ). Neither AMP nor ADP allosterically regulated SNF1 activity (data not shown), but ADP, and not AMP or ATP, protected SNF1 from dephosphorylation on Thr210 by recombinant protein phosphatase 2C (Figures 1A and 1B). The half-maximal effect of ADP in this assay was ∼500 μM (Figure 1A), which fits well with previous studies showing that total (extractable) ADP concentrations in yeast range from 0.4 to 1.5 mM (Canelas et al., 2008; Ditzelmüller et al., 1983; Larsson et al., 1997). ADP also produced a similar degree of protection when a heterotrimeric SNF1 complex containing the Sip2 β subunit isoform, rather than Gal83, was used in the dephosphorylation assay (Figure 1C). We have also shown the same effect of ADP using SNF1 complexes purified from yeast. We expressed a TAP-tagged form of Snf1 to purify the complex from cells grown on sucrose medium, a growth condition in which Snf1 is phosphorylated on Thr210. The purified enzyme could be efficiently dephosphorylated by treatment with recombinant PP2C (Figure 1D). Consistent with our results obtained using recombinant SNF1 expressed in *E. coli*, addition of ADP, but not AMP, resulted in significant protection of the enzyme from dephosphorylation. The protective effect of ADP occurred over a similar concentration range to that using recombinant SNF1 isolated from *E. coli*. Genetic studies have indicated that dephosphorylation of SNF1 in vivo is catalyzed by the yeast PP1 homolog Glc7. Importantly, we were also able to demonstrate that ADP protects against dephosphorylation of SNF1 by Glc7 (Figure 1E). ADP also inhibits dephosphorylation of SNF1 by rabbit PP1 (data not shown), strongly suggesting that the effect of ADP is on the SNF1 complex and not on the protein phosphatase per se.

### Adenine Nucleotides and NADH Bind to Snf4

**Figure 2:**
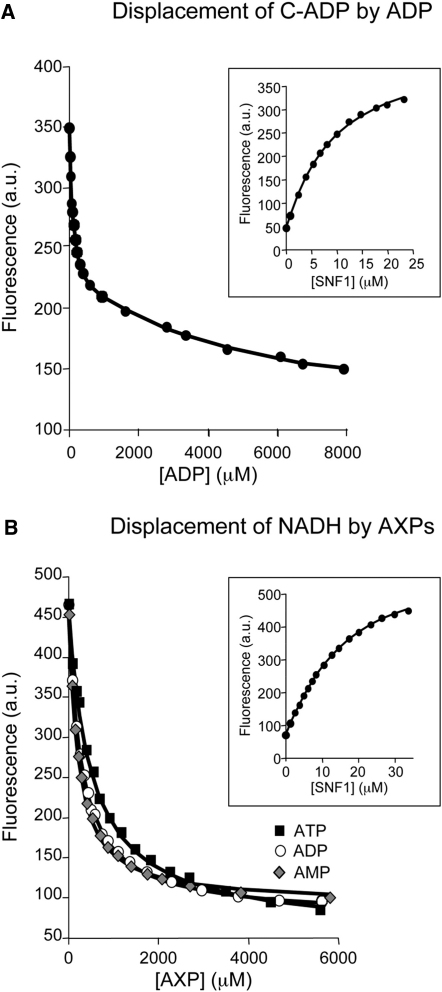
Measurement of Equilibrium Dissociation Constants for the Binding of AXPs to Phosphorylated SNF1 (A) Displacement of coumarin-ADP (C-ADP) from the SNF1:(C-ADP)_2_ complex by AXPs monitored using the change in fluorescence at 470 nm (excitation at 430 nm). The solid lines are the computed best fits with K_d,I_ and K_d,II_ for C-ADP binding fixed at 9 and 27 μM, respectively. Inset: titration of C-ADP with SNF1. (B) Displacement of NADH from the SNF1:NADH complex by AXPs monitored using the change in fluorescence at 435 nm (excitation at 340 nm). The solid lines are the computed best fits with the K_d_ for NADH fixed at 12.3 μM Inset: titration of NADH with SNF1. All measurements were carried out at 20°C in 50 mM Tris (pH 8), 100 mM NaCl, 1 mM tris(2-carboxyethyl)phosphine.

To understand better the regulation of SNF1 by ADP, we did a series of binding studies using the phosphorylated, active form of the enzyme (Figure 2 and Table 1). First, we examined nucleotide binding using analogs of ADP and ATP carrying a coumarin fluorescent reporter group in competition assays. These experiments revealed two distinct nucleotide binding sites; AMP, ADP, and ATP bind with similar affinities at a tight site (K_d,I_ for ADP ∼80 μM) and at a weaker one (K_d,II_ for ADP ∼800 μM). Under physiological conditions, most of the ATP and some of the ADP is complexed with magnesium. Therefore, we examined the effect of magnesium on binding of coumarin-AXPs to SNF1. The results show that magnesium weakens the binding of coumarin-AXP at both the strong and the weak sites (data shown in Table S4). Since ATP (and ADP) binding to SNF1 is significantly weaker than for the coumarin adducts, it is not possible to establish a robust estimate of the binding constant for Mg.ATP binding at the weaker site. But on the basis of the effect of magnesium on the binding of coumarin-ATP, binding of ATP to the weaker site is likely to be at least 5-fold weaker with magnesium than in its absence. As such, there is likely to be relatively little Mg.ATP (or non-Mg-bound ATP, of which there is little present within the cell) bound to the weak site of Snf4.

During the course of our studies, and in common with our recent studies on mammalian AMPK, we found that NADH undergoes a significant change in its fluorescence signal upon binding to the enzyme. We exploited this property to establish that NADH binds to the phosphorylated SNF1 complex with a stoichiometry of 1:1 and a dissociation constant of approximately 12 μM (obtained at 20°C, see Experimental Procedures). Intriguingly, NADH binding is specific in the sense that NAD^+^ binding is much weaker (K_d_ > 250 μM), presumably due to the presence of two positively charged arginine residues (Arg143 and Arg219) near the nicotinamide ring of NADH (see Figure S1). In further competition binding assays, we found that NADH competes with AXPs for their binding to just the tighter of the two sites (Table 1).

### Crystallization Studies Show that NADH Binds to Site 4 in Snf4

**Figure 3:**
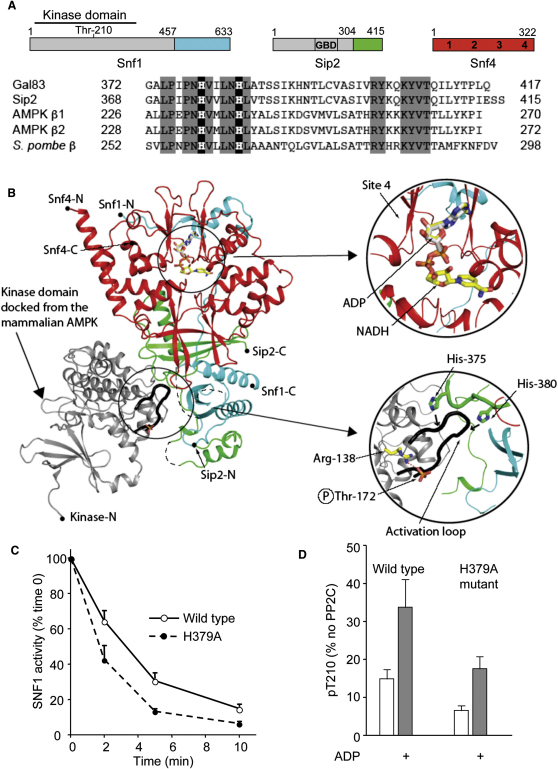
Mutation of His379 in Gal83 Increases the Rate of SNF1 Dephosphorylation (A) Schematic representation of the three components of the SNF1 heterotrimer from *S. cerevisiae*. The regions used for crystallization are shown in color, and the designation of the nucleotide binding sites within Snf4 corresponding to the 4 cystathionine-β-synthase domains, as proposed by Kemp et al. (Kemp et al., 2007), is indicated. An amino acid alignment of the C-terminal regions of Gal83, Sip2, human AMPKβ1, human AMPKβ2, and *S. pombe* AMPKβ subunits is also shown. Conserved residues are shown boxed in gray, and the two conserved histidine residues (that mediate the interaction between the kinase and the regulatory fragment in mammalian AMPK [Xiao et al., 2011]) are shown in black boxes. (B) Ribbon representation of the structure of the regulatory fragment determined in this study; the C-terminal domain of Snf1 is colored blue, the C-terminal domain of Sip2 is colored green, and the Snf4 subunit is colored red, with the active kinase domain of mammalian AMPK (colored in gray) docked onto the complex. The positions of both ADP and NADH, determined in this study by crystal soaking experiments, are shown in ball-and-stick representation. The upper inset panel on the right shows a closer view of ADP and NADH binding to site 4, while the lower inset panel shows the activation loop region of the kinase domain and two histidine residues from Sip2. (C) Dephosphorylation rate of wild-type or Gal83 His379 to alanine (H379A) SNF1 complex. (D) Protection of dephosphorylation of wild-type and H379A mutant by ADP (800 μM) after incubation with PP2C for 10 min. Results are the mean ± SEM for 3–4 independent experiments.

In order to discover the location of the tight nucleotide binding site on SNF1, we carried out AXP and NADH crystal soaking experiments. Using a tricistronic expression system, we expressed and purified a regulatory fragment of SNF1 from *S. cerevisiae* (Figure 3A) comprising the C-terminal domains of Snf1 (457–633) and Sip2 (304–415) together with full-length Snf4 (1–332). We collected native diffraction data extending to 2.1 Å Bragg spacing, from which the structure was solved by molecular replacement. Relevant crystallographic statistics are given in Table S1. A previous study (Amodeo et al., 2007) reported the structure of the regulatory core of SNF1 (comprising residues 398–633 of Snf1, 154–415 of Sip2, and 1–322 of Snf4). The overall architecture of the structure reported here corresponds closely with the previously reported structure (Figure S1), although the structure reported in our current study lacks the glycogen binding domain of Sip2. Data sets from soaked crystals show strong, well-resolved electron density for AMP, ADP, and NADH (Figures 3B and S1) at one of the four canonical nucleotide binding sites on Snf4 (labeled site 4 according to the convention suggested by Kemp [Kemp et al., 2007]). Given that NADH binds substantially more tightly to SNF1 than AXPs, and given the relative concentrations of AXPs and NADH in yeast (Canelas et al., 2008), we conclude that under most conditions this site will be occupied by NADH. The situation is therefore analogous to mammalian AMPK, where the equivalent site contains a nonexchangeable AMP (Xiao et al., 2007). We think it likely that nucleotide occupancy at this site, in both SNF1 and AMPK, is important for the stability of the enzyme, since protein stability experiments (Figure S2A) indicate that SNF1 is substantially more stable in the presence of NADH.

### ADP Binding at the Weaker Site Protects against Dephosphorylation of SNF1

Two lines of evidence lead us to conclude that binding of ADP at the weaker of the two binding sites is responsible for protection of the enzyme against dephosphorylation. First, as described above, NADH competes for the tighter nucleotide binding site, but has no effect on SNF1 activity or on the ability of ADP to protect against dephosphorylation (Figure S2B). In contrast, and as would be predicted, AMP, which binds at both nucleotide binding sites, competes with ADP in the dephosphorylation protection assay (Figure S2C). Second, the half-maximal effect of ADP on dephosphorylation of SNF1 occurred at a much higher concentration (>500 μM for recombinant SNF1) than the K_d_ for the tighter binding site (∼80 μM), but close to the K_d_ of the weaker site.

Presumably reflecting the weak binding constant and the lattice packing, we have been unable to obtain crystals of SNF1 with nucleotide present in the weaker site. As an alternative approach, we carried out extensive mutagenesis studies aimed at disrupting the individual nucleotide binding sites within Snf4, but, in common with our experience with mammalian AMPK, we have not been able to identify mutations that specifically ablate AXP or NADH binding (see Tables S2 and S3 and Figure S3). These data clearly show that mutation of the aspartic acid residues that are seen to interact with the ribose moiety of AXPs and NADH do not lead to the ablation of ligand binding. The notion that these aspartic acid residues were required for binding was first suggested by us to explain why one of the four “canonical” binding sites in AMPK did not bind AXPs (Xiao et al., 2007). However, our suggestion was untested at that time, and presumably the lack of effect of these mutations reflects the fact that the energetic costs of desolvating the carboxyl of the aspartic acid and the hydroxyls of the ribose offsets the contribution to binding of the hydrogen bonds that the aspartic acids make with AXPs. Importantly, however, in the absence of mutants that ablate AXP binding, our structural studies provide valuable insights into the likely mechanism of ADP-mediated protection against dephosphorylation.

### ADP Promotes Interaction of the T-Loop with the Regulatory Core of SNF1

We recently determined the structure of an active form of mammalian AMPK in which the T-loop, containing phosphorylated Thr172, is well ordered and interacts with the C-terminal domains of the α and β subunits (Xiao et al., 2011). In this conformation, Thr172 is protected from dephosphorylation because access to protein phosphatases is restricted. We showed that part of the linker between the N-terminal kinase and the C-terminal domain of the α subunit formed an α-hook structure that enhanced the association of the kinase domain with the regulatory fragment when ADP, but not ATP, is bound (Xiao et al., 2011). The sequences of the T-loop and the residues with which it interacts are highly conserved between the yeast and mammalian enzymes, but the sequences of the linker region are not (Xiao et al., 2007, 2011) (Figure 3A). His233 in AMPKβ1 (His235 in β2) lies at the interface of the regulatory domain with the kinase domain, and mutation of this residue in AMPK significantly increases the rate of dephosphorylation (Xiao et al., 2011). Mutation of His379 in Gal83 (corresponding to His233 in AMPKβ1 and His375 in Sip2; see Figure 3A) also significantly increased the rate of dephosphorylation of SNF1, while leaving protection by ADP intact (Figures 3C and 3D), as was also found for the equivalent mutation in AMPK (Xiao et al., 2011).

## Discussion

An essential role for SNF1 in glucose repression has been known for nearly 30 years (Carlson et al., 1981, 1984), and activation of SNF1 during glucose limitation has been appreciated for nearly 20 years (Woods et al., 1994), but the metabolic signal that triggers it has remained elusive. Here we show that ADP binds to Snf4 and protects SNF1 against dephosphorylation of Thr210, suggesting that ADP is the metabolic signal that leads to activation of SNF1 in response to glucose starvation in budding yeast. We recently showed that ADP protects mammalian AMPK against dephosphorylation (Xiao et al., 2011), and while our manuscript was under review, Oakhill et al. reported that ADP, as well as AMP, stimulates phosphorylation of AMPK on Thr172 (Oakhill et al., 2011). AMP and ADP stimulation of phosphorylation requires myristoylation of the β subunit of AMPK (Oakhill et al., 2010). However, Gal83, the predominant β isoform of *S. cerevisiae* SNF1, does not contain a consensus sequence for N-terminal myristoylation, suggesting that Gal83-containing SNF1 complexes are not subject to this form of regulation.

In mammalian AMPK, we showed that ADP protects against dephosphorylation by promoting the interaction of the T-loop region with the regulatory regions of the α and β subunits (Xiao et al., 2011). Our current study suggests that a similar mechanism operates for ADP-mediated protection against dephosphorylation of SNF1. However, the lack of sequence conservation between SNF1 and AMPK in the α-hook region (Xiao et al., 2011) means that we are less clear about the molecular mechanism for coupling nucleotide binding to the recruitment, and protection against dephosphorylation, of the kinase domain. Nonetheless, the fact that the interaction of the kinase domain with the regulatory fragment is highly asymmetric means that it must be one of the ADP binding sites on the surface of Snf4 that faces the kinase domain (labeled site 2 and site 3 in Figure S3) that mediates protection against dephosphorylation. Given that the linker region is not conserved between mammals and yeast, and that ADP has been shown to bind to site 2 in the *S. pombe* homolog of AMPK (Jin et al., 2007), we think it is likely that ADP binding at site 2 in Snf4 mediates the protective effect against dephosphorylation of SNF1. In this model we envisage that a region of Snf1 between the kinase domain and the ordered C-terminal domain, which is not conserved in AMPKα, plays a role analogous to the α-hook in AMPK. How this mechanism discriminates for ADP over AMP and ATP for protection against dephosphorylation is not clear. In order to address this, additional structures of full-length SNF1 containing the phosphorylated kinase domain, together with the Snf4 subunit in complex with AMP, ADP, and ATP, will be required. Importantly, the concentration of ADP required for protection of SNF1 against dephosphorylation in vitro is much higher than that required for mammalian AMPK, in keeping with the higher concentrations of ADP present in yeast cells relative to mammalian cells. Our results show that magnesium weakens the binding of ATP to Snf4, and therefore we would expect very little ATP to be bound at the regulatory site. Thus, in contrast to mammalian AMPK, where ADP competes for binding with Mg.ATP, SNF1 may only bind significant amounts of ADP. These data lead us to a conclusion similar to that reached by Shapiro and colleagues in their study of the AMPK homolog from *Schizosaccharomyces pombe*, where they could not identify magnesium in their ATP-bound crystal structure (Townley and Shapiro, 2007), leading them to suggest that the discrimination against magnesium-bound ATP would enable that enzyme to respond to the much lower levels of AMP in the presence of high competitive levels of total ATP. Given our demonstration that for *S. cerevisiae* the relevant ligand is ADP, and given its binding constant relative to Mg.ATP, we suggest that in *S. cerevisiae* SNF1 may respond to absolute changes in ADP concentration. Finally, the finding that ADP protects both yeast SNF1 and mammalian AMPK against dephosphorylation raises the exciting possibility that ADP might represent a “unifying trigger” for activation of AMPK orthologs in all species.

## Experimental Procedures

### Production of Recombinant SNF1

DNA encoding Snf1 and Snf4 were cloned into pRSFDuet (Novagen), and cDNA encoding Gal83 was cloned into pETDuet1; both were generous gifts from Marian Carlson (Columbia University). For expression of SNF1, *E. coli* BL21 (DE3) cells were cotransformed with these vectors, and the SNF1 complex was purified as described below. Tricistronic vectors expressing His-Snf1Sip2_(154-415)_Snf4 or His-Snf1_(457-633)_Sip2_(304-415)_Snf4 were constructed and used to transform *E. coli* BL21 (DE3) cells for expression of SNF1 complexes. Initially, SNF1 complexes were purified by affinity chromatography using nickel-Sepharose (Sanders et al., 2007) and used for further studies.

### Tandem Affinity Purification of Glc7 and Snf1

The Snf1 and Glc7 proteins were purified from yeast cell extracts using tandem affinity purification (TAP) method (Rigaut et al., 1999). For both genes, the TAP tag was added at the C terminus of the open reading frame. Low-copy number plasmids expressing the TAP constructs were introduced to cells lacking the endogenous chromosomal *SNF1* or *GLC7* gene. Cells were grown on media with sucrose or glucose as the carbon source, respectively. TAP-tagged proteins were purified as previously described for the Snf1-TAP protein (Elbing et al., 2006). Dephosphorylation reactions using Glc7 were performed immediately following purification, as the enzyme rapidly loses activity over time.

### SNF1 Functional Assays

Recombinant SNF1 complexes expressed in *E. coli* were phosphorylated by incubation with CaMKKβ as described previously (Sanders et al., 2007). An aliquot of the phosphorylated protein (∼1 μg) was incubated in 50 mM HEPES (pH 7.4), 2.5 mM MgCl_2_, and in the presence or absence of PP2Cα (26 ng) in the presence or absence of nucleotides (as stated in the figure legends) for 10 min at 37°C. The level of Thr210 phosphorylation was determined by western blot analysis using an anti-AMPK Thr172 phosphospecific antibody (Cell Signaling). Total Snf1 was detected using an anti-6xHis antibody (Abcam). In some cases, SNF1 activity was measured directly using the SAMS peptide assay (Woods et al., 1994). Dephosphorylation of TAP-SNF1 by recombinant PP2Cα was monitored using phosphopeptide-specific antiserum (McCartney and Schmidt, 2001), while the level of total Snf1 protein was determined with His-probe antibodies (Santa Cruz Biotechnology). Primary antibodies were detected using LI-COR IRDye Infrared Dye secondary antibodies and visualized using an Odyssey Infrared Imager (LI-COR Biotechnology). Quantification of results was performed using Odyssey software and expressed as a ratio of the signal obtained with the phosphospecific antibody relative to the appropriate total antibody.

### Binding Studies

Unless stated otherwise, all binding measurements were performed at 20°C in 50 mM Tris (pH 8), 100 mM NaCl, 1 mM tris(2-carboxyethyl)phosphine. Free NADH has an emission maximum at 465 nm (excitation at 340 nm). In the presence of saturating SNF1, the emission maximum is blue shifted to 435 nm, and the fluorescence emission intensity is increased. These fluorescence changes were used to determine equilibrium dissociation constants by titrating 4–10 μM NADH with SNF1. Experiments performed at higher NADH concentration and low temperature confirmed that NADH binds to SNF1 with 1:1 stoichiometry.

The coumarin analogs of ADP and ATP (C-ADP, C-ATP: 3′-(7-diethylaminocoumarin-3-carbonylamino)-3′-deoxyadenosine 5′ di- and triphosphate) have emission maxima at 479 nm (excitation at 430 nm). In the presence of saturating SNF1 the emission maxima are blue shifted to ∼470 nm and the fluorescence intensity is increased. These fluorescence changes were used to determine equilibrium dissociation constants by titrating 4–6 μM C-AXP with SNF1. Experiments performed at higher C-AXP concentration and low temperature confirmed that these analogs bind to SNF1 with 2:1 stoichiometry. Dissociation constants for NADH and C-AXPs to phosphorylated and nonphosphorylated SNF1 are shown in Table S4. Dissociation constants for AMP, ADP, and ATP were determined using NADH and the coumarin analogs as reporters. Competition assays were performed in which a mixture of SNF1 and the fluorescent nucleotide was titrated with the nonfluorescent nucleotide. These data were then analyzed with the previously determined K_d_ values for the fluorescent nucleotides held constant in the analysis. The dissociation constant for NAD^+^ was estimated using NADH as the reporter.

### Crystallographic Studies

Truncated SNF1 (Snf4_(1-322)_Sip2_(304-415)_His-Snf1_(457-633)_) was cloned into a tricistronic vector and subsequently expressed in *E. coli* BL21 (OneShot BL21 Star [DE3] cells, Invitrogen). Proteins were purified by nickel affinity chromatography (His-Trap, GE Healthcare), anion exchange (Mono Q, GE Healthcare), and gel filtration (Superdex 200, GE Healthcare). The His-tag was removed by digestion with PreScission protease, and the SNF1 complex protein was purified by gel filtration (Superdex 200, GE Healthcare). Protein complex stock solution was prepared at 15 mg/ml in 50 mM Tris (pH 7.0), 300 mM NaCl, and 1 mM TCEP. Crystals were grown by vapor diffusion technique at 18°C in hanging drops. Drops were prepared by mixing equal volumes of protein complex with reservoir solution containing 1.0 M succinic acid, 0.1 M HEPES (pH 7.0), 1% (w/v) polyethylene glycol monomethyl ether 2000 (Index Reagent 34, Hampton Research). Crystals were first transferred into mother liquor with an additional 12.5% ethylene glycol, then 25% ethylene glycol, prior to plunging into liquid nitrogen. For crystal soaking experiments, ADP, AMP, and NADH were made up in reservoir buffer, and crystals were soaked overnight prior to flash cooling. Diffraction data were collected on an in-house MicroMax-007 HF rotating anode coupled to a RaxisIV^2+^ detector. Data were integrated using Denzo and scaled with Scalepack (Otwinowski and Minor, 1993). The structure was solved by molecular replacement using Amore (Navaza, 1994) using 2QLV.pdb as the search model. Standard refinement was carried out with Refmac5 (CCP4, 1994) with manual model building with Coot. Figures were created with PyMOL.

## Figures and Tables

**Table 1:** Equilibrium Dissociation Constants for the Binding of AXPs to Phosphorylated SNF1

Ligand	K_d_ (μM)	K_d,I_ (μM)	K_d,II_ (μM)
Versus NADH	Versus C-AXPs	
AMP	55 (12)	62 (15)	1450 (350)
ADP	72 (14)	91 (25)	760 (200)
ATP	120 (25)	85 (22)	1200 (300)

Dissociation constants were determined by competition against NADH or 3′-(7-diethylaminocoumarin-3-carbonylamino)-3′-deoxy-AXP (C-AXP). The K_d_ values shown are the mean (±SD) determined from at least three independent measurements. All measurements were carried out at 20°C in 50 mM Tris (pH 8), 100 mM NaCl, 1 mM tris(2-carboxyethyl)phosphine. The methods employed in analyzing the fluorescence binding data are fully described elsewhere (Xiao et al., 2011).

## Accession Numbers

Coordinates have been deposited in the Protein Data Bank with accession codes 3TDH, 3T4N, and 3TE5.

## Supplemental Information

Document S1. Three Figures, Four Tables, and Supplemental References

## References

[bib1] Amodeo G.A., Rudolph M.J., Tong L. (2007). Crystal structure of the heterotrimer core of Saccharomyces cerevisiae AMPK homologue SNF1. Nature.

[bib2] Canelas A.B., van Gulik W.M., Heijnen J.J. (2008). Determination of the cytosolic free NAD/NADH ratio in Saccharomyces cerevisiae under steady-state and highly dynamic conditions. Biotechnol. Bioeng..

[bib3] Carling D. (2004). The AMP-activated protein kinase cascade—a unifying system for energy control. Trends Biochem. Sci..

[bib4] Carlson M., Osmond B.C., Botstein D. (1981). Mutants of yeast defective in sucrose utilization. Genetics.

[bib5] Carlson M., Osmond B.C., Neigeborn L., Botstein D. (1984). A suppressor of SNF1 mutations causes constitutive high-level invertase synthesis in yeast. Genetics.

[bib6] Celenza J.L., Carlson M. (1986). A yeast gene that is essential for release from glucose repression encodes a protein kinase. Science.

[bib7] CCP4 (Collaborative Computational Project, Number 4) (1994). The CCP4 suite: programs for protein crystallography. Acta Crystallogr. D Biol. Crystallogr..

[bib8] Davies S.P., Helps N.R., Cohen P.T., Hardie D.G. (1995). 5′-AMP inhibits dephosphorylation, as well as promoting phosphorylation, of the AMP-activated protein kinase. Studies using bacterially expressed human protein phosphatase-2C alpha and native bovine protein phosphatase-2AC. FEBS Lett..

[bib9] Ditzelmüller G., Wöhrer W., Kubicek C.P., Röhr M. (1983). Nucleotide pools of growing, synchronized and stressed cultures of *Saccharomyces cerevisiae*. Arch. Microbiol..

[bib10] Elbing K., Rubenstein E.M., McCartney R.R., Schmidt M.C. (2006). Subunits of the Snf1 kinase heterotrimer show interdependence for association and activity. J. Biol. Chem..

[bib11] Gancedo J.M. (1992). Carbon catabolite repression in yeast. Eur. J. Biochem..

[bib12] Hardie D.G. (2007). AMP-activated/SNF1 protein kinases: conserved guardians of cellular energy. Nat. Rev. Mol. Cell Biol..

[bib13] Hardie D.G., Carling D., Carlson M. (1998). The AMP-activated/SNF1 protein kinase subfamily: metabolic sensors of the eukaryotic cell?. Annu. Rev. Biochem..

[bib14] Hong S.P., Leiper F.C., Woods A., Carling D., Carlson M. (2003). Activation of yeast Snf1 and mammalian AMP-activated protein kinase by upstream kinases. Proc. Natl. Acad. Sci. USA.

[bib15] Jin X., Townley R., Shapiro L. (2007). Structural insight into AMPK regulation: ADP comes into play. Structure.

[bib16] Kahn B.B., Alquier T., Carling D., Hardie D.G. (2005). AMP-activated protein kinase: ancient energy gauge provides clues to modern understanding of metabolism. Cell Metab..

[bib17] Kemp B.E., Oakhill J.S., Scott J.W. (2007). AMPK structure and regulation from three angles. Structure.

[bib18] Lage R., Diéguez C., Vidal-Puig A., López M. (2008). AMPK: a metabolic gauge regulating whole-body energy homeostasis. Trends Mol. Med..

[bib19] Larsson C., Nilsson A., Blomberg A., Gustafsson L. (1997). Glycolytic flux is conditionally correlated with ATP concentration in *Saccharomyces cerevisiae*: a chemostat study under carbon- or nitrogen-limiting conditions. J. Bacteriol..

[bib20] McCartney R.R., Schmidt M.C. (2001). Regulation of Snf1 kinase. Activation requires phosphorylation of threonine 210 by an upstream kinase as well as a distinct step mediated by the Snf4 subunit. J. Biol. Chem..

[bib21] Mitchelhill K.I., Stapleton D., Gao G., House C., Michell B., Katsis F., Witters L.A., Kemp B.E. (1994). Mammalian AMP-activated protein kinase shares structural and functional homology with the catalytic domain of yeast Snf1 protein kinase. J. Biol. Chem..

[bib22] Navaza J. (1994). AMoRe: an automated package for molecular replacement. Acta. Crystallogr..

[bib23] Oakhill J.S., Chen Z.P., Scott J.W., Steel R., Castelli L.A., Ling N., Macaulay S.L., Kemp B.E. (2010). β-Subunit myristoylation is the gatekeeper for initiating metabolic stress sensing by AMP-activated protein kinase (AMPK). Proc. Natl. Sci. Acad. USA.

[bib24] Oakhill J.S., Steel R., Chen Z.P., Scott J.W., Ling N., Tam S., Kemp B.E. (2011). AMPK is a direct adenylate charge-regulated protein kinase. Science.

[bib25] Otwinowski Z., Minor W., Sawyer L., Isaacs N., Bailey S. (1993). Data Collection and Processing.

[bib26] Rigaut G., Shevchenko A., Rutz B., Wilm M., Mann M., Séraphin B. (1999). A generic protein purification method for protein complex characterization and proteome exploration. Nat. Biotechnol..

[bib27] Rubenstein E.M., McCartney R.R., Zhang C., Shokat K.M., Shirra M.K., Arndt K.M., Schmidt M.C. (2008). Access denied: Snf1 activation loop phosphorylation is controlled by availability of the phosphorylated threonine 210 to the PP1 phosphatase. J. Biol. Chem..

[bib28] Sanders M.J., Grondin P.O., Hegarty B.D., Snowden M.A., Carling D. (2007). Investigating the mechanism for AMP activation of the AMP-activated protein kinase cascade. Biochem. J..

[bib29] Suter M., Riek U., Tuerk R., Schlattner U., Wallimann T., Neumann D. (2006). Dissecting the role of 5′-AMP for allosteric stimulation, activation, and deactivation of AMP-activated protein kinase. J. Biol. Chem..

[bib30] Sutherland C.M., Hawley S.A., McCartney R.R., Leech A., Stark M.J.R., Schmidt M.C., Hardie D.G. (2003). Elm1p is one of three upstream kinases for the Saccharomyces cerevisiae SNF1 complex. Curr. Biol..

[bib31] Townley R., Shapiro L. (2007). Crystal structures of the adenylate sensor from fission yeast AMP-activated protein kinase. Science.

[bib32] Wilson W.A., Hawley S.A., Hardie D.G. (1996). Glucose repression/derepression in budding yeast: SNF1 protein kinase is activated by phosphorylation under derepressing conditions, and this correlates with a high AMP:ATP ratio. Curr. Biol..

[bib33] Woods A., Munday M.R., Scott J., Yang X., Carlson M., Carling D. (1994). Yeast SNF1 is functionally related to mammalian AMP-activated protein kinase and regulates acetyl-CoA carboxylase in vivo. J. Biol. Chem..

[bib34] Xiao B., Heath R., Saiu P., Leiper F.C., Leone P., Jing C., Walker P.A., Haire L., Eccleston J.F., Davis C.T. (2007). Structural basis for AMP binding to mammalian AMP-activated protein kinase. Nature.

[bib35] Xiao B., Sanders M.J., Underwood E., Heath R., Mayer F.V., Carmena D.J., Jing C., Walker P.A., Eccleston J.F., Haire L.F. (2011). Structure of mammalian AMPK and its regulation by ADP. Nature.

[bib36] Zhang B.B., Zhou G., Li C. (2009). AMPK: an emerging drug target for diabetes and the metabolic syndrome. Cell Metab..

