# Supplementary material for: ADP Regulates SNF1, the Saccharomyces cerevisiae Homolog of AMP-Activated Protein Kinase
Source: Cell Metab. 2011 Nov 2;14(5):707–14. doi: 10.1016/j.cmet.2011.09.009 (PMC3241989; doi:10.1016/j.cmet.2011.09.009)
Supplement: Document S1. Three Figures, Four Tables, and Supplemental References [file mmc1.pdf]

Supplemental Information

Cell Metabolism, *Volume 14*

ADP Regulates SNF1, the *Saccharomyces cerevisiae* Homolog of AMP-Activated Protein Kinase

Faith V. Mayer, Richard Heath, Elizabeth Underwood, Matthew J. Sanders, David Carmena, Rhonda R. McCartney, Fiona C. Leiper, Bing Xiao, Chun Jing, Philip A. Walker, Lesley F. Haire, Roksana Ogrodowicz, Stephen R. Martin, Martin C. Schmidt, Steven J. Gamblin, and David Carling

Figure S1

This shows additional structural information that is linked to the binding data (Figure 2 in main text). This Figure also includes an overlay of the structure determined in the current study with that of a previously reported structure (requested by one of the reviewers in the original submission).

Figure S2

This shows the effect of NADH binding on SNF1. The data relates to Figure 2 in the main text, but is also relevant to Figure 1 since we show that NADH has no direct effect on SNF1 activity.

Figure S3

This shows the effect of mutagenesis of residues within Snf4 on activity and nucleotide binding. We also show the location of bound nucleotides determined from our study, and also from a previous study on S. pombe SNF1. The data relate to Figure 3 in the main text as well as in the Discussion Section.

Table S1

Crystallographic statistics for the structural data (refers to Figure 3 within main text).

Table S2

Dissociation constants for nucleotide binding to SNF1 harbouring mutations within Snf4. This information was requested by one of the reviewers in the original submission.

Table S3

Dissociation constants for nucleotide binding to AMPK harbouring a mutation within γ1.

Tables 2 and 3 contain important information that is linked to the Discussion of the main text, as well as data in Figures 2 and 3.

Table S4

Dissociation constants for NADH and C-AXPs binding to SNF1. This information relates to the Experimental Procedures section.

Supplemental Figure S1

A

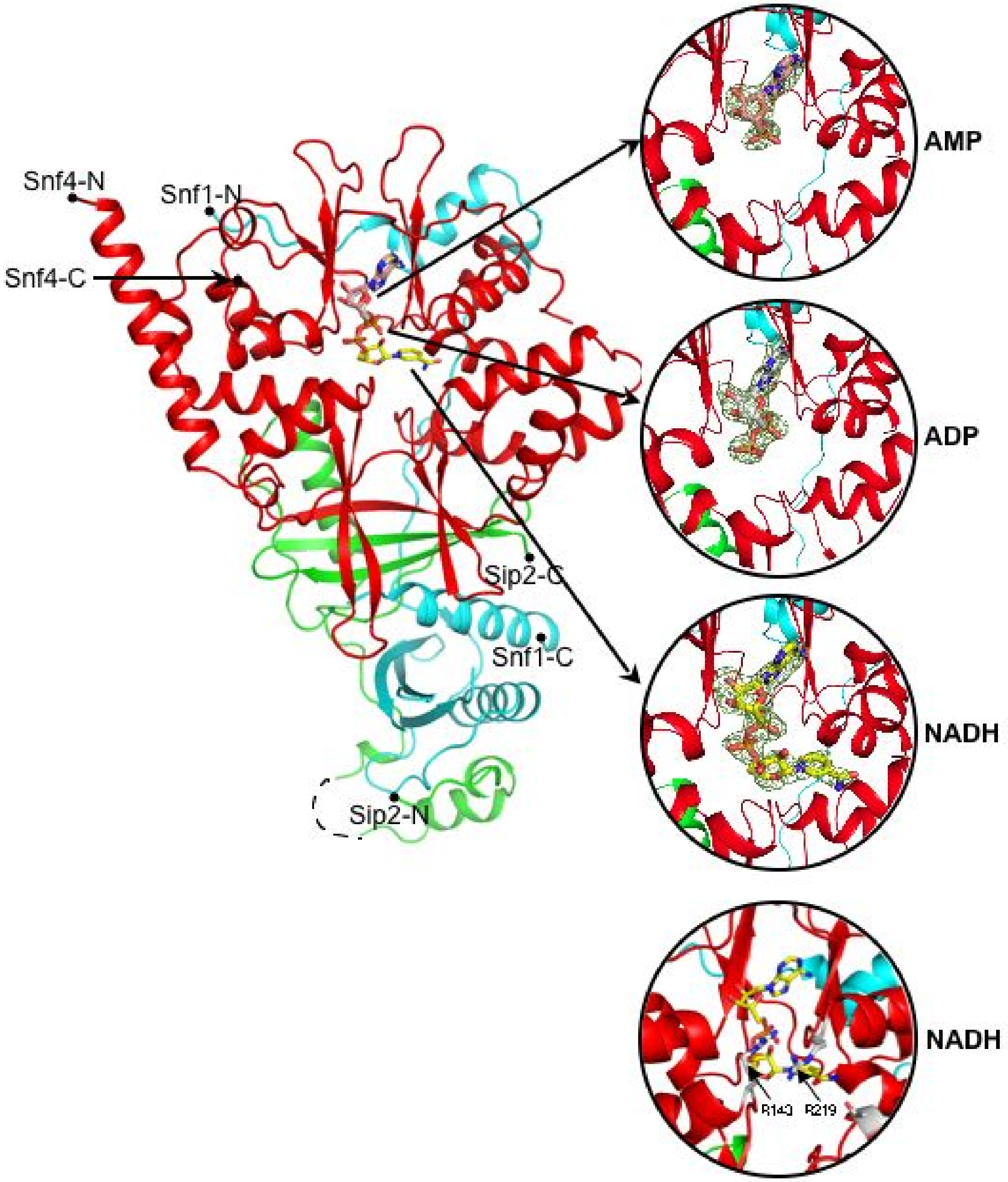

Supplemental Figure S1

B

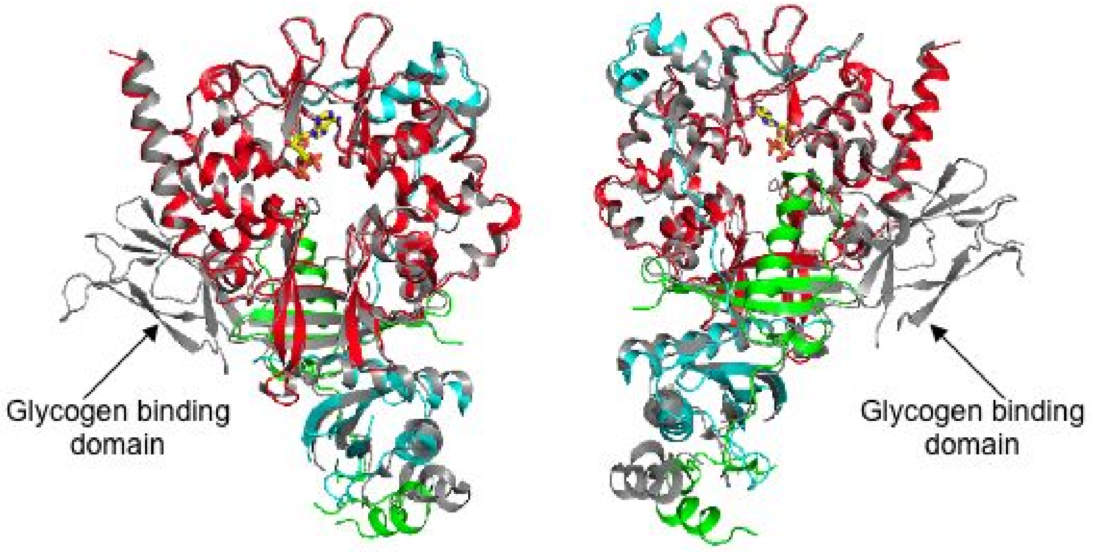

C

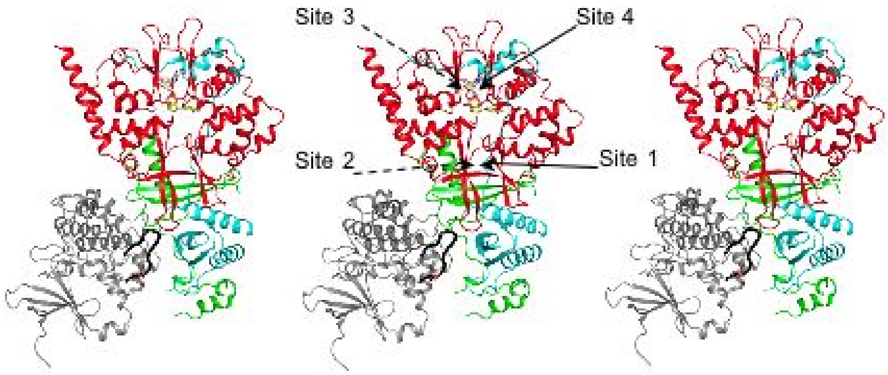

Supplemental Figure S1

D

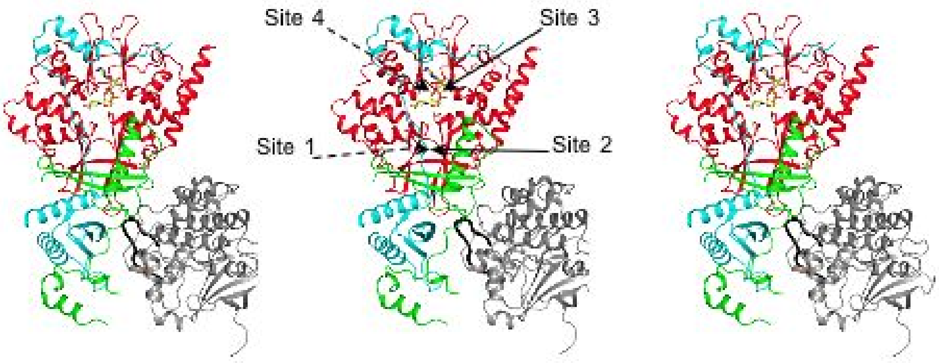

E

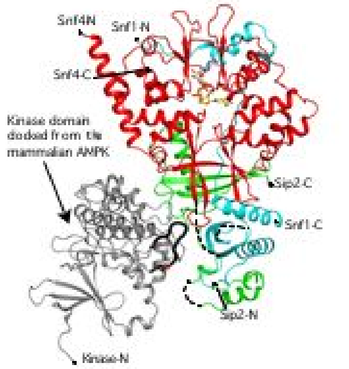

Figure S1. Crystal Structure of SNF1 with One Molecule of AMP, ADP or NADH Bound and Comparison with a Previously Reported Structure of SNF1

(A) Ribbon representation of the structure of the regulatory fragment of the SNF1 complex with AMP (pink), ADP (white), and NADH (yellow) bound at site 4. The electron density from Fo-Fc omit maps, where the nucleotide has been removed from the phasing model, is shown for each of the bound nucleotides. An additional panel highlights two arginine residues near the nicotinamide ring of NADH whose presence presumably accounts for the much weaker binding of NAD^+ to SNF1. The crystallographic statistics of the three nucleotide-bound structures are given in Supplementary Table 1.

(B) Superposition of the structure determined in the current study with the structure of the regulatory core of SNF1 (shown in grey) determined in a previous study (Amodeo et al., 2007) shown in two orthogonal views. In the current study, the region of Sip2 encoding the glycogen binding domain is not present in the construct used for crystallisation studies.

(C) Stereo representation of Figure 3B from the main manuscript. The left and middle panels are displayed as wall-eye stereo, and the middle and right panels displayed as cross-eyed.

(D) The same stereo representation as (C) after the complex has been rotated 180° about a vertical axis.

(E) A mono ribbon representation of the complex shown in (C) and (D) with the domains labelled.

Supplemental Figure S2

A

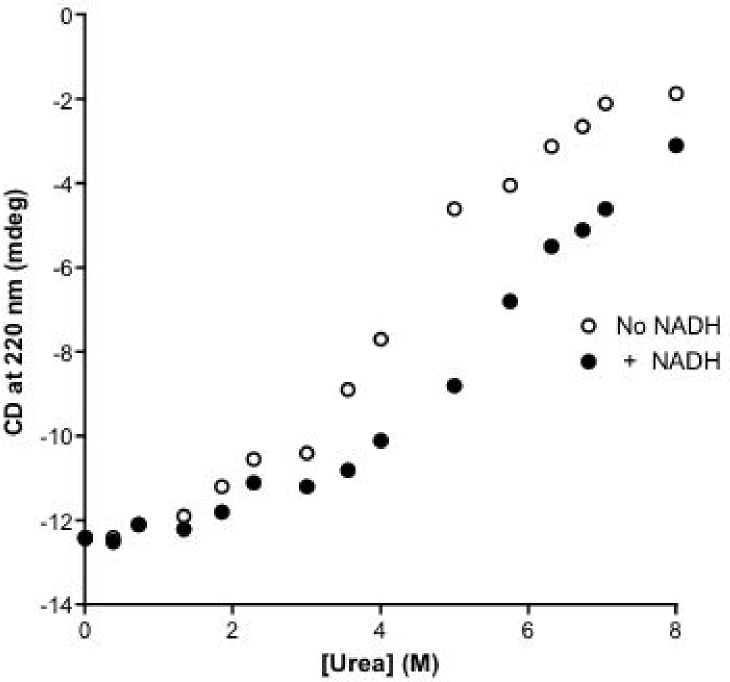

B

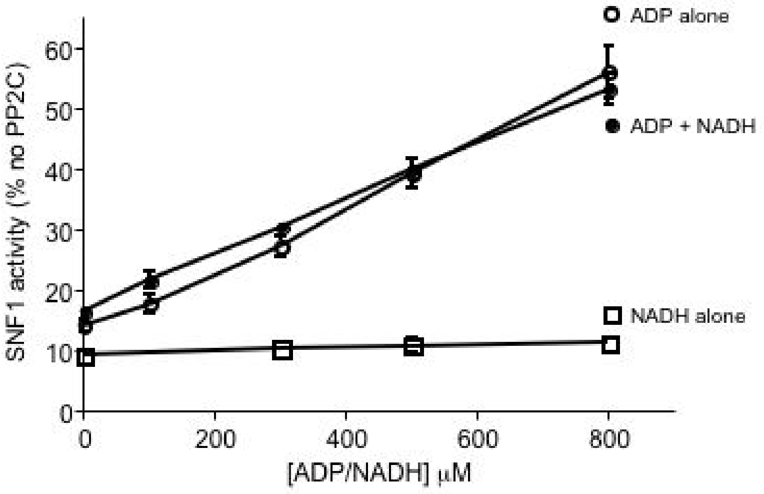

Supplemental Figure S2

C

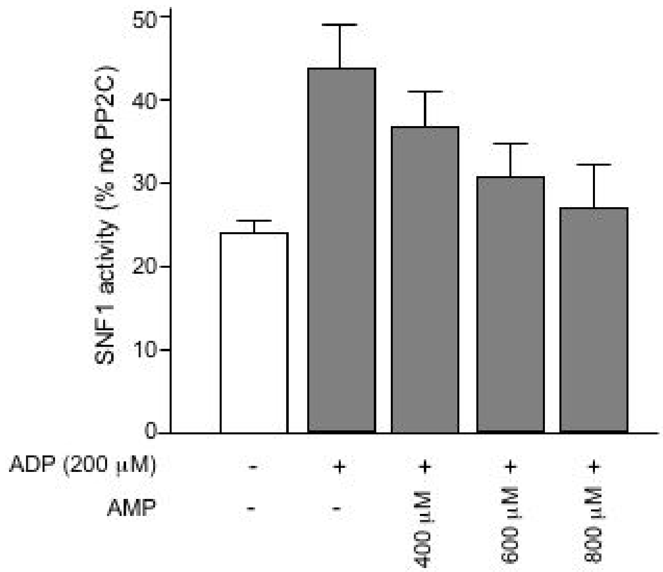

Figure S2. Effect of NADH Binding to SNF1

(A) Phosphorylated SNF1 complex (0.16 mg/ml) in 25 mM Tris, pH8, 0.5 mM tris(2-carboxyethyl)phosphine was incubated with varying concentrations of urea in the absence (open symbols) or presence (closed symbols) of NADH (0.1 mM). Protein unfolding was monitored using far UV circular dichroism.

(B) Recombinant SNF1 was phosphorylated with CaMKKβ and incubated for 10 min at 37°C with PP2C in the presence or absence of varying concentrations of ADP or NADH, or in the presence of 0.5 mM NADH and varying concentrations of ADP.

(C) Phosphorylated SNF1 was incubated for 10 min at 37°C with PP2C in the presence or absence of 200 μM ADP and increasing concentrations of AMP (400, 600 and 800 μM). In each case, an aliquot of the incubation mix was removed and diluted 20-fold with 50 mM HEPES, pH 7.4, 1 mM EDTA, 1 mM dithiothreitol. Where required, AMP, ADP and/or NADH were added to bring their final concentration to 40 μM in each case. SNF1 activity was measured using the SAMS peptide assay (Woods et al., 1994). Results are plotted as a percentage of the activity measured in the absence of PP2C and are the mean ±SEM of 3-4 independent experiments.

Supplemental Figure S3

A

```
Snf4     FLNSKTSYDVLPVSYRLIVLDTSLLVKKSLNVLLQNSIVSAPLWDSKTSRFAGLLTTTDF     84
AMPKγ1   FMKSHRCYDLIPTSSKLVVFDTSLQVKKAFFALVTNGVRAAPLWDSKKQSFVGMLTITDF     90

Snf4     INVIQYYFSNPDKFELVDKLQLDGLKDIERALGVDQLDT----ASIHPSRPLFEACLKML    140
AMPKγ1   INILHRYY----KSALVQIYELEEHK-IETWREVYLQDSFKPLVCISPNASLFDAVSSLI    145

Snf4     ESRSGRIPLIDQDEETHREIVVSVLTQYRILKFVALNCRE---THFLKIPIGDLNIITQD    197
AMPKγ1   RNKIHRLPVIDPESGN----TLYILTHKRILKFLKLFITEFPKPEFMSKSLEELQIGTYA    201

Snf4     NMKSCQMTTPVIDVIQMLTQGRVSSVPIIDENGYLINVYEAYDVLGLIKGGIYNDLSLSV    257
AMPKγ1   NIAMVRTTTPVYVALGIFVQHRVSALPVVDEKGRVVDIYSKFDVINLAAEKTYNNLDVSV    261

Snf4     GEALMRRSDDFEGVYTCTKNDKLSTIMDNIRKARVHRFFVVDDVGRLVGVLTLSDILKYI    317
AMPKγ1   TKALQHRSHYFEGVLKCYLHETLEAIINRLVEAEVHRLVVVDEHDVVKGIVSLSDILQAL    321

Snf4     LL  319
AMPKγ1   VL  323
```

B

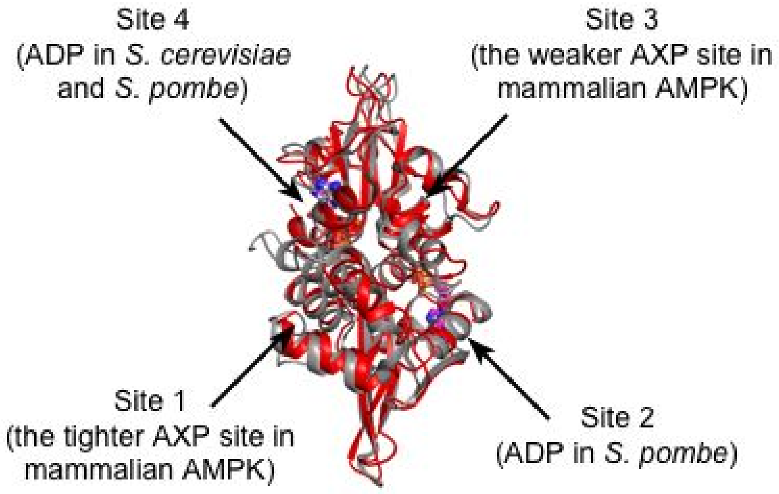

Supplemental Figure S3

C

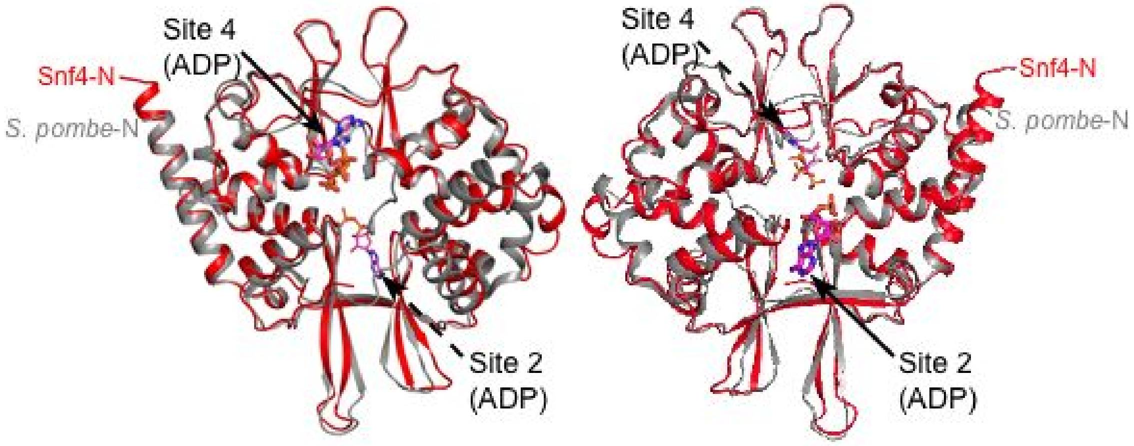

D

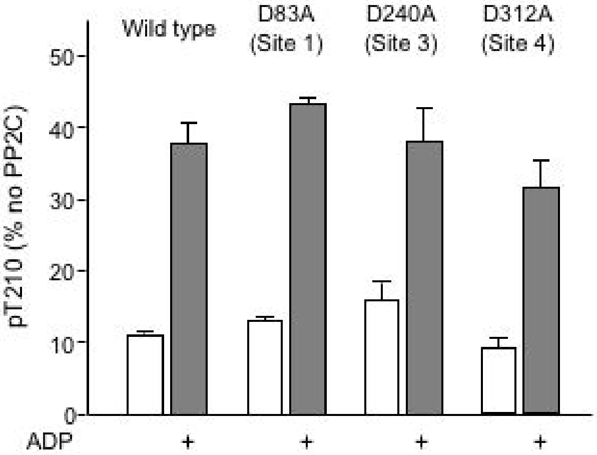

Figure S3. Mutagenesis Studies on the Canonical Nucleotide Binding Sites in SNF1

Since mutation of the aspartic acid residues that interact with the ribose of AXPs have been reported to have functional effects in mammalian AMPK (Oakhill et al., 2010) we made the equivalent mutations in SNF1.

(A) Amino acid alignment of Snf4 (*S. cerevisiae*) and AMPKγ1 (rat). Conserved residues are shown boxed in grey, and the aspartic acid residues that we previously identified as interacting with the hydroxyl groups on the ribose ring of bound nucleotide in site 1, site 3 or site 4 of AMPKγ1 (Xiao et al., 2007), are shown in black boxes. In site 2 an arginine residue (R169 in Snf4 and R170 in γ1) is present and this is also shown for reference. The N-terminal 24 amino acids of Snf4 and the N-terminal 30 amino acids of γ1 are not shown.

(B, C) Structural overlap of *S. cerevisiae* Snf4 and *S. pombe* γ subunits. Snf4 is shown in red and the *S. pombe* γ subunit in grey. The ADP molecules that are bound to *S. pombe* γ (Jin et al., 2007) are shown in magenta, whereas the ADP bound in Snf4 is shown in white. The subunits are shown "edge" on in panel (B) and in two orthogonal views in (C).

(D) Wild type SNF1 (Snf1Gal83Snf4) and SNF1 harbouring mutation of Asp83 to alanine in Snf4 (D83A in site 1), Asp240 to alanine (D240A in site 3) or Asp312 to alanine (D312A in site 4) were phosphorylated by CaMKKβ and incubated for 10 min at 37°C with PP2C in the presence or absence of ADP (800 μM). SNF1 activity was measured using the SAMS peptide and results are plotted as the mean ±S.E.M of 3 independent experiments. As can be seen, none of these mutations had any significant effect on ADP protection of dephosphorylation. We therefore undertook to characterise the nucleotide binding properties of these mutants (Supplementary Table 2).

Table S1. Crystallographic Statistics for Truncated SNF1 Complex with AMP, ADP and NADH Bound

	SNF1-AMP	SNF1-ADP	SNF1-NADH
Data Collection			
Space group	$C222_1$	$C222_1$	$C222_1$
Unit cell (Å)	a=95.3, b=242.2, c=79.2	a=89, b=251.2, c=79.8	a=95.9, b=241.8, c=79.4
Resolution (Å) (high res)	30-2.3 (2.4-2.3)	30-2.3 (2.4-2.3)	30-2.5 (2.6-2.5)
Rsym %	7.5 (37)	3.6 (21.3)	5.3 (31.4)
Completeness	100 (99.9)	96.2 (89.2)	100 (100)
Redundancy	5.6 (5.4)	3.8 (2.9)	7.1 (5.4)
Refinement			
Number of reflectio (work/free)	44315/2357	42698/2284	38804/2061
R_{fac} % (work/free)	24.6/26.6	23.6/26.4	24.7/28.1
Rms bonds/angles (°)	0.01/1.00	0.01/1.00	0.01/1.05
PDB entry code	1XYZ	2XYZ	3XYZ

Values in parentheses refer to the highest resolution shell.

$R_{sym} = \Sigma_j|\langle I\rangle - I_j|/\Sigma\langle I\rangle$ where I_j is the intensity of the jth reflection and $\langle I\rangle$ is the average intensity.

$R_{work} = \Sigma \mid |Fo| - |Fc| \mid / \Sigma|Fo|$.

$R_{free} = \Sigma_T \mid |Fo| - |Fc| \mid / \Sigma_T |Fo|$, where T is a test data set of 5% of the total reflections randomly chosen and set aside before refinement.

Table S2. Dissociation Constants for Binding of NADH and Coumarin-ADP to Wild-Type SNF1 Complex and SNF1 Harbouring Aspartic Acid to Alanine Mutations in Sites 1, 3 and 4 of Snf4

	NADH	Coumarin-ADP	
Snf4 protein	K_d (μM)	$K_{d,I}$ (μM)	$K_{d,II}$ (μM)
Wild-type	18.7 (3.8)	4.2 (0.4)	14.7 (4.9)
D83A (site 1)	75.7 (29.9)	1.8 (0.7)	24.9 (4.9)
D240A (site 3)	14.3 (3.7)	2.6 (1)	45.3 (19)
D312A (site 4)	45 (4.2)	8.4 (4.1)	38.9 (5.6)

The K_d values shown are the mean (±SD) from 3 (NADH) or 2 independent experiments (coumarin-ADP). All measurements were carried out using truncated SNF1 complex (the same complex used for our crystallographic studies). For NADH binding, samples were incubated at 20°C in 50 mM Tris, pH 8, 150 mM NaCl, 1 mM tris(2-carboxyethyl)phosphine, and for coumarin-ADP binding, samples incubated at 7°C in 50 mM Tris, pH 8, 1 mM tris(2-carboxyethyl)phosphine.

Table S3. Dissociation Constants for the Binding of AXPs to Wild-Type and D89A AMPK

In order to determine whether the lack of abolishment of nucleotide binding by the aspartic acid to alanine mutations in SNF1 was maintained in AMPK we examined in detail the properties of an equivalent mutation in AMPKγ1. Aspartic acid residue 89 interacts with nucleotide bound in site 1 (the tighter of the two exchangeable binding sites in AMPK, and the site that we have previously shown binds NADH (Xiao et al., 2011)). We mutated this aspartic acid residue to alanine (D89A) and determined nucleotide binding in the mutant AMPK complex compared to wild type AMPK by competition experiments using NADH. As summarised in the table below, the D89A mutation did not significantly affect nucleotide binding at site 1.

Ligand	K_d (μM)	K_d (μM)
	vs NADH	
	**Wild type**	**D89A mutant**
AMP	1.65 (0.40)	0.95 (0.25)
ADP	1.15 (0.35)	1.25 (0.25)
ATP	0.75 (0.25)	1.40 (0.40)

Wild type and D89A were determined at 7°C using methods previously described (Xiao et al., 2011). The lower temperature was used here in order to permit more accurate determination of the dissociation constants for NADH binding (18.5 ± 2.5 μM for the wild type and 7.1 ± 1.2 μM for D89A (mean ±SD)).

Therefore, although the mutant has effects on both the allosteric activation, and protection against dephosphorylation of the enzyme, these two effects cannot be attributed to ablation of nucleotide binding at site 1. To try to overcome this problem we have designed and made a number of other mutants in all four of the canonical AXP binding sites of SNF that target the adenine nucleotide binding pocket. To date, however, we have not been able to identify mutants that specifically ablate nucleotide binding.

Table S4. Dissociation Constants for NADH and C-AXPs Binding to SNF1

Ligand	K_d (μM)	$K_{d,I}$ (μM)	$K_{d,II}$ (μM)
	**Phosphorylated SNF1**		
NADH	12.3 (4)	--	--
C-ADP	--	9 (3)	27 (9)
C-ATP	--	12 (5)	31 (7)
C-ADP + Mg^{2+}		45.1 (16.8)	95.9 (32.6)
C-ATP + Mg^{2+}		41.6 (3.6)	n.d.
	**Non-phosphorylated SNF1**		
NADH	5.6 (1.6)	--	--
C-ADP	--	7.5 (3)	23 (8)
C-ATP	--	10.0 (4.5)	21 (7)

The K_d values shown are the mean (±SD) from at least 3 independent experiments (2 independent experiments for binding in presence of magnesium). All measurements were carried out at 20°C in 50 mM Tris, pH 8, 100 mM NaCl, 1 mM tris(2-carboxyethyl)phosphine in the presence or absence of 5 mM $MgCl_2$. A value for the Kd of C-ATP in the presence of magnesium could not be determined accurately (n.d.).

Supplemental References

Amodeo, G., Rudolph, M.J., and Tong, L. (2007). Crystal structure of the heterotrimer core of Saccharomyces cerevisiae AMPK homologue SNF1. Nature 449, 492-495.

Jin, X., Townley, R., and Shapiro, L. (2007). Structural Insight into AMPK Regulation: ADP Comes into Play. Structure 15, 1285-1295.

Oakhill, J.S., Chen, Z.P., Scott, J.W., Steel, R., Castelli, L.A., Ling, N., Macaulay, S.L., and Kemp, B.E. (2010). Beta subunit myristoylation is the gatekeeper for initaiting metabolic stress sensing by AMP-activated protein kinase (AMPK). Proc. Natl. Sci. Acad. USA 107, 19237-19241.

Woods, A., Munday, M.R., Scott, J., Yang, X., Carlson, M., and Carling, D. (1994). Yeast SNF1 is functionally related to mammalian AMP-activated protein kinase and regulates acetyl-CoA carboxylase in vivo. J Biol Chem 269, 19509-19515.

Xiao, B., Heath, R., Saiu, P., F.C., L., Leone, P., Jing, C., Walker, P.A., Haire, L., Eccleston, J.F., Davis, C.T., Martin, S.R., Carling, D., and Gamblin, S.J. (2007). Structural basis for AMP binding to mammalian AMP-activated protein kinase. Nature 449, 496-500.

Xiao, B., Sanders, M.J., Underwood, E., Heath, R., Mayer, F., Carmena, D.J., Jing, C., Walker, P.A., Eccleston, J.E., Haire, L.F., Martin, S.R., Carling, D., and Gamblin, S.J. (2011). Structure of mammalian AMPK and its regulation by ADP. Nature 472, 230-233.
